# Age-Related Changes in Posture Steadiness in the Companion Dog

**DOI:** 10.1093/geroni/igab046.3461

**Published:** 2021-12-17

**Authors:** Alejandra Mondino Vero, Grant Wagner, Edgar Lobaton, Katharine Russell, Natasha Olby

**Affiliations:** 1 North Carolina State University, Raleigh, North Carolina, United States; 2 NCSU, NCSU, North Carolina, United States; 3 North Carolina State University, NCSU, North Carolina, United States

## Abstract

Aging is associated with changes in the sensory-motor system that could lead to dynamic instability. In fact, postural control deficits have been proposed as an early indicator of frailty. Measurements of the displacement of the center of pressure (COP) using pressure mat data are useful tools to determine postural steadiness. Companion dogs represent a powerful model to study aging in people because they share our environment and experience similar age-related diseases. To date, the effect of aging on postural control in dogs has not yet been evaluated. The aim of this study was to determine the correlation between age and the displacement of the COP in dogs during quiet standing. Due to the diversity of life expectancy in dogs according to their body size, age was normalized as a fraction of the predicted life expectancy. Dogs older than 75% of their life expectancy (n=18) were asked to stand on a pressure mat for 8 seconds per trial during at least five trials. Only the frames where the dogs were standing still and facing forward were analyzed. Age as a fraction of life expectancy was significantly correlated (p<0.05) with the Medio-lateral Range, Root-Mean-Square Distance, 95% Confidence Ellipse, and Total Sway Area of the COP. These results show that, as in humans, aging in dogs is associated with postural control deficits and therefore reinforce the dog as a suitable model for translational studies of aging and postural steadiness.

